# Seasonality of Polyaromatic Hydrocarbons (PAHs) and Their Derivatives in PM_2.5_ from Ljubljana, Combustion Aerosol Source Apportionment, and Cytotoxicity of Selected Nitrated Polyaromatic Hydrocarbons (NPAHs)

**DOI:** 10.3390/toxics11060518

**Published:** 2023-06-08

**Authors:** Ivana Drventić, Mateo Glumac, Ivana Carev, Ana Kroflič

**Affiliations:** 1 Department of Analytical Chemistry, National Institute of Chemistry, Hajdrihova 19, 1000 Ljubljana, Slovenia; ivana.drventic@ki.si; 2 Faculty of Chemistry and Chemical Technology, University of Ljubljana, Večna pot 113, 1000 Ljubljana, Slovenia; 3 Laboratory for Cancer Research, School of Medicine, University of Split, Šoltanska 2, 21000 Split, Croatia; mateo.glumac@mefst.hr; 4 NAOS Institute of Life Science, 355 rue Pierre-Simon Laplace, 13290 Aix-en-Provence, France; carev.ivana@gmail.com; 5 Mediterranean Institute for Life Science, Meštrovićevo šetalište 45, 21000 Split, Croatia; 6 Faculty of Science, University of Split, Ruđera Boškovića 33, 21000 Split, Croatia; 7 Department of Catalysis and Chemical Reaction Engineering, National Institute of Chemistry, Hajdrihova 19, 1000 Ljubljana, Slovenia

**Keywords:** fine particulate matter, PAH diagnostic ratios, PM sources, incomplete combustion aerosols, NPAH cytotoxicity, 1-nitropyrene (1-nP), 9-nitroanthracene (9-nA), 6-nitrochrysene (6-nC), 6-nitrobenzo[a]pyrene (6-nBaP)

## Abstract

Airborne particulate matter (PM) is a vector of many toxic pollutants, including polyaromatic hydrocarbons (PAHs) and their derivatives. Especially harmful is the fine fraction (PM_2.5_), which penetrates deep into the lungs during inhalation and causes various diseases. Amongst PM_2.5_ components with toxic potential are nitrated PAHs (NPAHs), knowledge of which is still rudimentary. Three of the measured NPAHs (1-nitropyrene (1-nP), 9-nitroanthracene (9-nA), and 6-nitrochrysene (6-nC)) were detected in ambient PM_2.5_ from Ljubljana, Slovenia, along with thirteen non-nitrated PAHs. The highest concentrations of pollutants, which are closely linked with incomplete combustion, were observed in the cold part of the year, whereas the concentrations of NPAHs were roughly an order of magnitude lower than those of PAHs throughout the year. Further on, we have evaluated the toxicity of four NPAHs, including 6-nitrobenzo[a]pyrene (6-nBaP), to the human kidney cell line, HEK293T. The most potent was 1-nP (IC_50_ = 28.7 µM), followed by the other three NPAHs, whose IC_50_ was above 400 or 800 µM. According to our cytotoxicity assessment, atmospheric 1-nP is the most harmful NPAH among the investigated ones. Despite low airborne concentrations of NPAHs in ambient air, they are generally considered harmful to human health. Therefore, systematic toxicological assessment of NPAHs at different trophic levels, starting with cytotoxicity testing, is necessary in order to accurately evaluate their threat and adopt appropriate abatement strategies.

## 1. Introduction

To protect the environment and human health, air-quality-monitoring programs exist all over the world, setting limit values for the most harmful air pollutants. Annual mean concentration of the toxic and high-priority polyaromatic hydrocarbon (PAH), benzo[a]pyrene (BaP), is set at 1 ng m^−3^ air (for its total content in PM_10_, i.e., particulate matter (PM) that is smaller than 10 μm; EU Directive 2004/107/EC). However, no regulation or legislation exists to date controlling PAH content in the fine PM fraction (i.e., PM smaller than 2.5 μm, denoted PM_2.5_) or referring to the most critical nitrated and oxygenated PAH analogs (NPAHs and OPAHs), although they can be even more harmful than their parent PAHs [1]. Therefore, in recent years, the occurrence and concentration of PAH derivatives have especially been topical issues in atmospheric research [2,3]. 

Other, less frequent preventive measures comprise a combination of air-quality-monitoring and source apportionment studies in which major sources of airborne pollutants are identified so that they can be more efficiently mitigated in a targeted way. Although PAHs and NPAHs can be of natural origin, their anthropogenic sources, such as the combustion of fossil fuels, largely prevail in urbanized areas [4]. Most NPAHs, including the four target ones in this study, i.e., 1-nitropyrene (1-nP), 9-nitroanthracene (9-nA), 6-nitrochrysene (6-nC), and 6-nitrobenzo[a]pyrene (6-nBaP), are considered primary pollutants and are directly emitted in the air during incomplete combustion of different fuels (diesel, gasoline, coal, biomass, and others) [5,6]. Amongst many different approaches to identify sources of PAH pollution that have been applied to date [7,8,9,10], diagnostic ratios turned out to be a simple yet often unreliable tool to distinguish between airborne pollution originating from petroleum spills, fuel combustion, and coal or biomass burning [11]. 

Nevertheless, every air-quality-monitoring and source apportionment study starts by applying robust, accurate, and possibly simple analytical methods to ambient samples. For the determination of PAHs and their derivatives, chromatographic techniques are usually employed. Air quality management has already established a method for the determination of total PAHs in ambient PM_10_ (SIST EN 15549 and SIST ISO 12884); however, a standard method for substituted PAHs does not exist. The analysis of particulate pollutants typically starts with extraction, which is laborious, requires organic solvents that are harmful to the environment and human health [12], and as a multi-step process quickly induces errors [13]. In recent years, however, many world-class environmental chemists have focused on developing greener methods that will facilitate trace PM analyses, including for PAHs and their derivatives.

It has been generally accepted that most of the PAHs are toxic, and some exhibit carcinogenic and mutagenic properties [14,15]. The primary mechanism of PAH-induced toxicity is through the formation of reactive oxygen species, whereas other mechanisms also include inflammation, cell toxicity, mutagenicity, genotoxicity, teratogenicity, and carcinogenicity [5]. On the other hand, NPAHs and OPAHs could cause especially severe adverse effects on the environment and human health [3,5]. While toxic equivalency factors (TEFs), which are used to express human toxicity of a pollutant in terms of BaP, are already available for many unsubstituted PAHs; TEFs for NPAHs are often missing [16]. Although NPAHs have rarely been tested even on human cell lines [17], their toxic potential is theoretically huge due to the aromatic nitro groups [5,18]. Previous studies have demonstrated much higher TEFs of some NPAHs compared to their parent PAHs [19,20]. However, as the toxicity of nitroaromatic compounds generally depends on various structural features (e.g., number and topology of nitro-groups, presence of other structural fragments) [21], it is not easy to predict the severity of adverse effects nitrated aromatic pollutants will cause both on the level of cells and tissues/organs. 

As typical constituents of fine airborne particles, NPAHs can penetrate deep into the lungs after inhalation where they also accumulate [22,23,24]. Consequently, the most pronounced effect on human health is on the respiratory system, causing asthma and allergies [25,26], obstructive pulmonary disease [27], and even lung cancer [28,29,30,31]. Despite poor water solubility, PAHs and their metabolites have been found in blood, urine, and breast milk, pointing to their absorption into the human body [5,32]. Moreover, differential accumulation of PAHs has been reported in sturgeons from the Caspian Sea, with the highest concentrations found in the kidneys and liver [33]. Air pollution has been in general associated with various human diseases for a long time, including neurological diseases [34], digestive system diseases [35], and cardiovascular problems [36,37]. 

We measured seasonal concentrations of thirteen priority PAHs and three NPAHs in ambient PM_2.5_ collected in Ljubljana, Slovenia. Unfortunately, 6-nBaP could not be determined by our new method based on thermal desorption gas chromatography with electron ionization mass spectroscopic detection (TD/GC-MS) [38], which was for the first time applied to a large set of ambient samples. The developed TD/GC-MS method, compared to the conventional method, allows for the direct analysis of PM samples circumventing complex extraction procedures, which results in labor savings and protection of the environment (no waste produced). Based on the obtained results, seasonal variation of thirteen PAHs and three NPAHs is presented for an urban environment in Central Europe. The measured concentrations were among others correlated with black carbon (BC) emissions that have been apportioned to traffic and biomass-burning sources in our recent work [39] to assess the contribution of distinct primary combustion sources to particulate PAHs and NPAHs in the atmosphere. We further combined those correlations with diagnostic ratios between specific PAHs and confirmed the attribution of major PM sources from our previous study [39]. Cytotoxicity of the selected four NPAHs was further evaluated on human embryonic kidney cells (HEK293T), commonly used non-tumor cells for cytotoxic evaluation of nanoparticles and PAHs [40,41]. Thus far, only 1-nP has been tested on a human endothelial cell line isolated from the umbilical cord vein, i.e., HUVEC cells [17]. Such basic cytotoxicity assessment is usually used as the first step toward toxicological studies on animals and humans.

## 2. Materials and Methods

### 2.1. Standards and Stock Solutions

A certified standard mixture for PAHs, Supelco^®^ EPA 525 PAH Mix B at 500 μg mL^−1^ each component in acetone contains acenaphthylene (Acy), anthracene (Ant), benz[a]anthracene (BaA), benzo[b]fluoranthene (Bbf), benzo[k]fluoranthene (Bkf), benzo[ghi]perylene (BgP), benzo[a]pyrene (BaP), chrysene (Cry), dibenz[a,h]anthracene (DBA), fluorene (Flu), indeno[1,2,3-c,d]pyrene (Ind), phenanthrene (Phe), and pyrene (Pyr).

The following BCR^®^ certified standard materials were used for the preparation of stock solutions of NPAHs, which were of similar concentrations to the PAH mix: 9-nA, 6-nC, 1-nP, and 6-nBaP. Each purchased solid standard was dissolved in acetone (GC-MS grade) to obtain 500 μg mL^−1^ and stored in a freezer at −20 °C before use. For cytotoxicity studies, stock NPAH solutions of different concentrations were prepared in DMSO: 1-nP (100 mM), 6-nC (50 mM), 6-nBaP (50 mM), and 9-nA (100 mM).

### 2.2. Sample Collection and Sampling Site

Sixty-five 24-h PM_2.5_ samples were collected on quartz fibre filters (Pall, 47 mm diameter) on a terrace of the National Institute of Chemistry in Ljubljana, Slovenia from August 2020 to July 2021 using a low-volume PM sampler (Giano, Dado lab, constant flow of 2.3 m^3^ h^−1^). Before and after sampling, the filters were conditioned for at least 24 h at constant temperature (22 ± 1 °C) and humidity (50 ± 5%) and weighted on a high-precision microbalance. After weighing for the second time (after the sampling), the filters were packed airtight and stored in a freezer at −20 °C until TD/GC-MS analysis.

In parallel, Aethalometer^®^ AE33 which measures PM light absorption at seven different wavelengths was used for online light-absorbing aerosol measurements. A numerical model was applied afterwards to identify contributions of traffic and biomass burning to the measured BC. The data are taken from our previous paper [39], and only daily averages are shown in this work.

Ljubljana is the capital of Slovenia with almost 294,000 inhabitants and extensive daily migrations. The city lies in a basin and is surrounded by a road ring. A significant amount of heat in the city is produced in a power and heat plant located about 5 km from the campaign site, mainly by natural gas, brown coal, and wood chips combustion. The monitoring site at the National Institute of Chemistry is located in a calm residential neighborhood and is mainly surrounded by individual houses and other research institutes. The district is close to one of the main roads leading to the city (500 m) and the city center itself (approx. 1 km).

### 2.3. TD/GC-MS Analysis

All samples were cut to 1/8 and directly analyzed by a GC (Agilent Technologies: 7890B GC System) equipped with a TD unit (Gerstel, TD3.5+) and a cooled injection system (CIS) (Gerstel, CIS4), and coupled to a single quadrupole MS (Agilent Technologies: 5977B MSD). The same amount of a filter sample, i.e., 1/8 filter was taken for the analysis every time. Prior to the analysis, no sample pre-treatment (extraction or similar) was applied. A non-polar HP-5 ms column ((5%-phenyl)-methylpolysiloxane, 30 m × 0.25 mm × 0.25 μm) was used for the separation of PAHs and NPAHs in ambient PM. The method and corresponding instrument parameters are described in detail elsewhere [38]. Only briefly, the analytes were thermally desorbed from ambient PM filters at 60 °C min^−1^ heating rate from 30 to 320 °C, which was kept for the next 12 min at 100 mL min^−1^ helium gas flow rate. Analyte trapping in CIS was assured at 5 °C, and injection to the GC column was carried out at 6 mL min^−1^ gas flow rate and 720 °C min^−1^ temperature ramp to 275 °C followed by 7.5 min heating at 275 °C. Chromatographic conditions were as follows. The column oven temperature program started at 55 °C, where it was initially held for 7 min, followed by a temperature increase to 170 °C at 25 °C min^−1^ ramp and further increase to 325 °C at 15 °C min^−1^. The final temperature of 325 °C was held for 3 min. Selected ion-monitoring (SIM) mode was used to detect and quantify ultra-trace PAHs and NPAHs in ambient PM samples. Peak identification and target ion selection were based on a standard mix (Supelco^®^ EPA PAH Mix B and BCR^®^ certified standard materials for NPAHs). The method was applicable to all target analytes except for 6-nBaP, which could not be sufficiently desorbed from the filter matrix due to its extremely high boiling point (524.1 °C at 760 mmHg compared to 402.9, 505, and 445.5 for 9-nA, 6-nC, and 1-nP, respectively).

### 2.4. Cell Culture, Treatments, and Cell Counting

Human embryonic kidney cells (HEK293T) were obtained from the German Collection of Microorganisms and Cell Cultures (DSMZ) and cultured in Dulbecco’s modified Eagle’s growth medium (D6429, Sigma-Aldrich) with 10% fetal bovine serum (F0804, Sigma-Aldrich) and 100 U of penicillin/streptomycin (P0781, Sigma-Aldrich) in a humidified incubator at 37 °C and 5% CO_2_ atmosphere. Trypsin-EDTA buffer (T4049, Sigma-Aldrich) was used for cell passaging and division. For every experiment, 5 × 10^4^ cells were seeded into 24-well plates 24 h before treatment with increasing concentrations of NPAHs, as indicated in Table S1. A single standard compound was always dissolved in DMSO before it was added to the cell culture. An equivalent amount of DMSO as used in the highest-concentration treatment was used for treating control cells in parallel. Cells were exposed to the selected pollutants for 24 h and then detached from the plate by trypsinization. Floating cells were separated from the growth medium by centrifugation, washed, and resuspended in PBS. Trypan blue staining (T8154, Sigma) was used for viability testing. Using a Neubauer chamber, cell counting was performed on an Olympus CHX41 microscope (Olympus Corporation).

### 2.5. Statistical Analysis

The cytotoxicity results were analyzed using GraphPad Prism (version 9). Normalized cell count data were fitted using the [Inhibitor] vs. normalized response-variable slope in-built equation in the software. All plotted curves were accompanied by profile likelihood intervals and showed good fit (R > 0.95). The results are provided as best-fit IC_50_ values.

Experiments containing three or more groups with a single variable were analyzed using one-way ANOVA. Post hoc analysis was performed using Tukey’s multiple comparison tests between two groups. For many-variable experiments, two-way ANOVA was applied and Dunnett’s multiple comparison tests were used for the post hoc analysis. Only the results with the calculated probability value (p) below 0.05 (*p* < 0.05) are considered significant. Symbols for different test significance levels are assigned as follows: not significant (ns) for *p* ≥ 0.05, * for 0.01 ≤ *p* < 0.05, ** for 0.001 ≤ *p* < 0.01, *** for 0.0001 ≤ *p* < 0.001, and **** for *p* < 0.0001. All data are presented as mean ± standard deviation (SD). The sample size was *n* = 3, containing biological replicates.

## 3. Results and Discussion

### 3.1. Seasonal Variability of PAHs and NPAHs

Seasonal airborne concentrations of fine particulate PAHs and NPAHs in Ljubljana, Slovenia measured by the direct TD/GC-MS method from August 2020 to July 2021 are given in Table S2. Particulate ∑PAH and ∑NPAH profiles are additionally shown in Figure S1. Cumulative median concentrations (min–max) of measured PAHs and NPAHs attached to PM_2.5_ in summer, autumn, winter, and spring were 0.208 (0.043–3.122) ng m^−3^, 8.807 (1.516–15.285) ng m^−3^, 9.234 (1.518–15.831) ng m^−3^, and 0.516 (0.050–2.584) ng m^−3^ for PAHs and 0.008 (0.001–0.063) ng m^−3^, 0.284 (0.153–0.706) ng m^−3^, 0.347 (0.079–0.545) ng m^−3^, and 0.005 (0.001–0.053 ng m^−3^ for NPAHs, respectively (refer here to Table 1).

**Figure 1:**
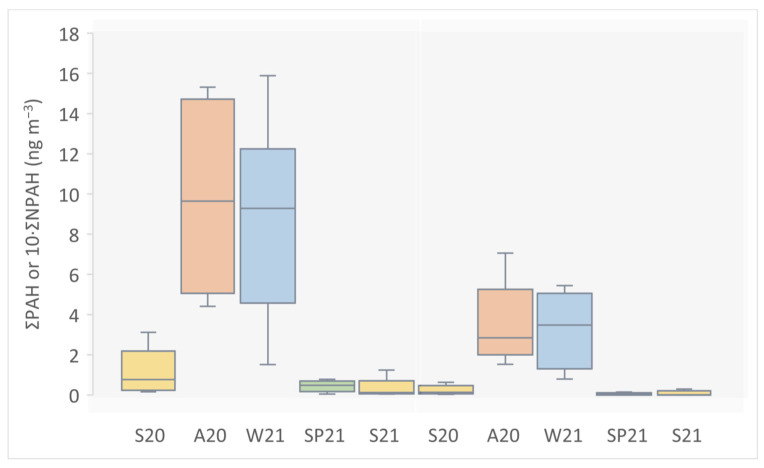
Cumulative air concentrations of measured PAHs (ΣPAH, left) and NPAHs (ΣNPAH, right) in PM_2.5_ from Ljubljana, Slovenia in different seasons: summer 2020 (S20), autumn 2020 (A20), winter 2021 (W21), spring 2021 (SP21), and summer 2021 (S21). Median, Q1, Q3, min and max concentrations of measured PAHs and NPAHs are shown.

The concentrations of NPAHs were generally an order of magnitude lower than those of PAHs (Figure 1 and Figure S1) and are comparable with previous measurements in Ljubljana and other Central European cities [1,42,43]. The three-ring PAHs are an exception, including Ant, whose concentration was even for an order of magnitude lower than the concentration of its corresponding 9-nA. This can be attributed to the relatively volatile character of Ant and three-ring PAHs in general, which results in their preferential partitioning to the atmospheric gas [44]. In general, low molecular weight PAHs with high vapor pressures are mainly distributed in the gas phase, while high molecular weight PAHs with low vapor pressures are predominantly found in the particulate phase [45,46]. Intermediate PAHs partition between both phases, and their phase distribution follows a typical seasonal pattern, i.e., in summer, higher gas-phase concentrations are expected due to volatilization at high temperatures [45,46]. Although it has been suggested to account for cumulative gas and particulate concentrations when assessing the atmospheric fate and related health risks of PAHs, including their nitrated and oxygenated derivatives [1], gas-phase concentrations are not in the scope of this study. Time series of selected PAH vs. NPAH pairs are additionally shown in Figure S2 (note the different scales), confirming predomination of all other parent PAHs over their analogous NPAHs in the particulate air fraction in all seasons.

The lowest ∑ (PAH + NPAH) was measured on 4 July 2021 (0.049 ng m^−3^), and the highest cumulative pollutant concentration was measured on 26 February 2021 (16.300 ng m^−3^), which resembles a typical seasonal pattern (Figure 1 and Figure S1). Significantly higher concentrations of primary PAHs and NPAHs in autumn and winter compared to late spring and summer can be attributed to (i) the use of biomass (and possibly coal) for household heating, which is confirmed in the following section, (ii) meteorological conditions that lower atmospheric boundary layer height in colder months, especially during the instances of temperature inversion typical of Ljubljana basin, (iii) moved partitioning equilibrium towards the particulate phase in colder months, and (iv) reduced photochemical decomposition in winter.

### 3.2. Pollution Sources of Measured PAHs and NPAHs

**Figure 2:**
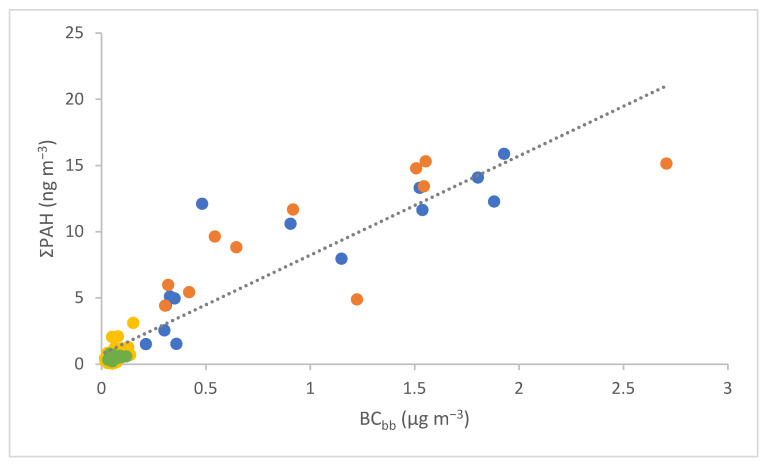
Positive correlation between cumulative particulate PAHs (ΣPAH) and black carbon from biomass burning (BC_bb_) for all four seasons. Yellow (summer) and green symbols (spring) present warm seasons (R^2^ = 0.3599), while cold seasons are depicted with orange (autumn, R^2^ = 0.5961) and blue symbols (winter, R^2^ = 0.755).

The measured PAH and NPAH concentrations were further correlated with daily BC concentrations previously attributed to fossil-fuel (BC_ff_) and biomass-burning emissions (BC_bb_) [39]. The best agreement was obtained between ΣPAH and BC_bb_ (Figure 2), which implies biomass burning is an important source of PAHs in the city, especially in colder months. Moreover, both ∑PAH and ∑NPAH were better correlated with the total BC than with BC_ff_ (see Figure S3), which connects both groups of compounds with other combustion emission sources besides traffic. In the case of NPAHs, for which even the correlation with BC_bb_ was not that evident (Figure S3), non-combustion or secondary processes could play a role, which is indicated by the changed ΣNPAH/ΣPAH ratio in the warm compared with the cold season. One would expect, however, that the ratio would have increased in the summer months due to photochemistry leading to the formation of NPAHs from their precursor PAHs [47]. Nevertheless, all investigated compounds in this work are typical primary pollutants; the reduced ΣNPAH/ΣPAH ratio most likely implying faster photodegradation of selected NPAHs compared to PAHs in sunny months.

Although the connection between the measured PAHs and primary emissions has been shown, the composition and concentration profiles (fingerprint) of combustion emissions are not unique to differentiate easily between distinct combustion sources [48]. For instance, emission factors are characteristic of the fuel burnt but are also closely connected with combustion conditions. Moreover, atmospheric removal and transformation processes affect different PAHs differently; therefore, their relative concentrations are not conserved over time. Considering also the semi-volatile character of the investigated compounds, which significantly affects partitioning equilibria at different atmospheric conditions, diagnostic ratios between specific PAHs are often not reliable enough to determine the origin of PAHs solely on this basis [9,49]. However, although it is widely accepted that diagnostic ratios are only applicable to receptor sites strongly affected by specific sources, they can still be useful, e.g., when considered together with more comprehensive receptor models.

**Figure 3:**
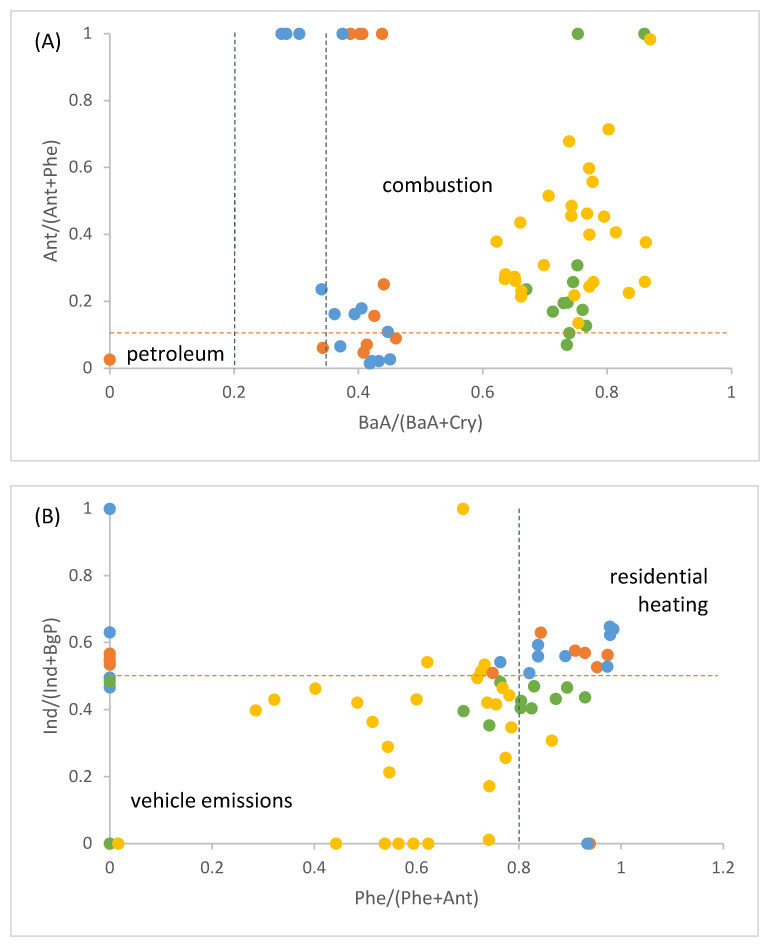
Diagnostic ratio plots to distinguish between (**A**) petroleum and combustion emission sources and (**B**) vehicle emissions and residential heating sources of ambient PAHs. Cold-season samples are depicted with orange (autumn) and blue symbols (winter) and warm-season samples with yellow (summer) and green symbols (spring). Threshold values are taken from Biache et al. [50], Galarneau et al. [9], and Tobiszewski and Namieśnik [11].

Figure 3A confirms combustion as a predominant source of particulate PAHs in Ljubljana. Except for one outlier, all other samples fall within the plot area characteristic of different combustion emissions, whereas warm- and cold-season samples cluster in two distinct regions. The cold-season samples were more characteristic of diesel exhaust, while warm-season samples lay in the region of gasoline emissions [47]. The latter could be due to the denser transient transport in a touristic season.

Air pollution due to residential heating is further distinguished from vehicle emissions in Figure 3B. The cold-season samples again cluster together within the area of residential heating, while the warm-season samples are much more spread over the vehicle emissions part of the plot. Those data points closer to the cold-season cluster mainly correspond to spring samples, possibly indicating an influence of open biomass burning due to agricultural activities.

Recently, positive matrix factorization (PMF) analysis has been applied to the same set of filter samples [39]. Six main sources of PM pollution were identified, including traffic emissions that were relatively constant throughout the year and the cold-season biomass-burning source, which was linked with individual heating devices. This supports our conclusions based on PAH diagnostic ratios. Additionally, in agreement with our observations, biomass-burning emissions were found to be characteristic of the spring samples [39]. Specific markers of coal combustion were not detected in either of the studies.

### 3.3. Cytotoxic Activity on Human Cell Lines

**Figure 4:**
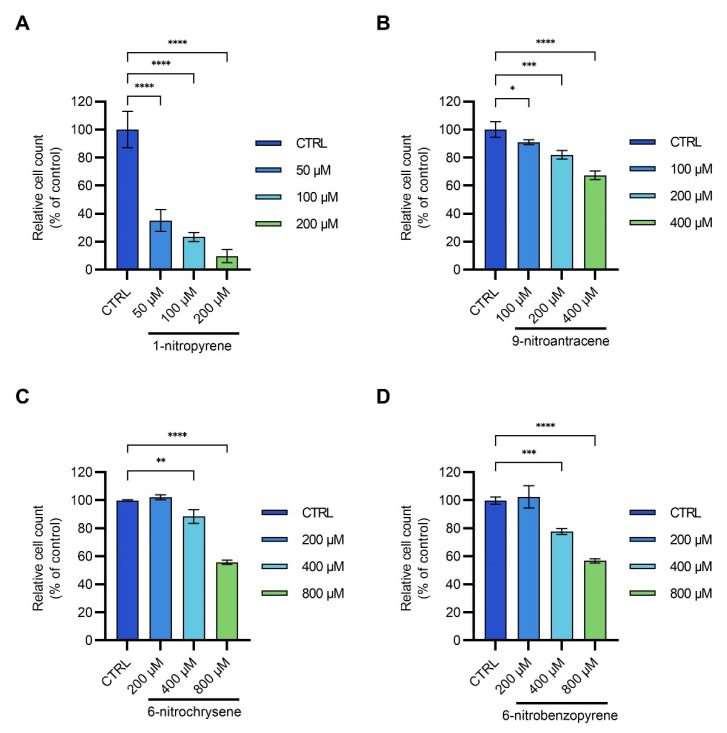
Relative cell counts after 24-h treatment of HEK293T cells with (**A**) 1-nitropyrene (1-nP), (**B**) 9-nitroanthracene (9-nA), (**C**) 6-nitrochrysene (6-nC), and (**D**) 6-nitrobenzo[a]pyrene (6-nBaP); for every treatment, control experiment (CTRL) is also given for comparison. Every experiment was performed in triplicate and statistically evaluated by Dunnett’s multiple comparison test. Data are shown as mean ± SD; different significance levels are assigned with asterisks: 0.01 ≤ *p* < 0.05 (*), 0.001 ≤ *p* < 0.01 (**), 0.0001 ≤ *p* < 0.001 (***), *p* < 0.0001 (****).

Human cell toxicity of the four NPAHs was evaluated on the human embryonic kidney cell line. Cell viability remained unaffected in any of the control groups treated with DMSO only. The cytotoxic effect was first evaluated by cell counting after 24 h treatment with the selected NPAHs (Figure 4). The most potent NPAH was 1-nP, reducing cell count already at concentrations below 50 µM (IC_50_ = 28.7 µM). This result is comparable with the previous study in which 1-nP reduced the umbilical cord cell count at a concentration of 15 µM after 24 h [17]. The other investigated NPAHS, 9-nA, 6-nC, and 6-nBaP, were much less potent than 1-nP. In the case of 9-nA, cell count reduction compared to the control was observed at a concentration around 100 µM. In comparison, 6-nC and 6-nBaP showed the effect only at 400 µM, and none reduced cell count below 50% compared to the control in the investigated concentration range (IC_50_ > 400 or 800 µM). The corresponding IC_50_ plots are given in Figure S4.

Besides total cell count, the number of dead cells after the exposure was also investigated (Figure S5). Among the tested compounds, 1-nP was again most effective, significantly inducing cell death already at 50 µM (*p* = 0.0258) followed by 6-nC at 200 µM (*p* = 0.0351), while 9-nA (*p* = 0.0002) and 6-nBaP (*p* = 0.0468) significantly induced cell death only at 400 µM. It is interesting to note that the proportion between the determined IC_50_ for 1-nP and 9-nA is similar as expressed in terms of TEF values, which refer to the toxicity on the level of the whole organism [1]. On the other hand, the highest potency of 6-nC in terms of TEF values among the tested compounds [1] was not confirmed by our results, which can be due to different damaging mechanisms involved that were out of scope of this study.

The observed inhibition of cell proliferation and induction of cell death can be explained by multiple mechanisms. It has been previously shown that engine emissions [50,51] and airborne particles [52,53] induce DNA damage in a form of DNA breaks and DNA adducts. Damage to DNA can lead to the inhibition of cell cycle progression and finally cause cell death [54,55]. On the other hand, cells that did not successfully repair DNA damage and somehow avoided cell death would acquire DNA mutations, accumulation of which increases susceptibility for the development of cancer [55,56]. It has been previously shown that 1-nP and 9-nA cause DNA mutations, which can lead to cancer [5,15,17,20,53,57,58].

**Figure 5:**
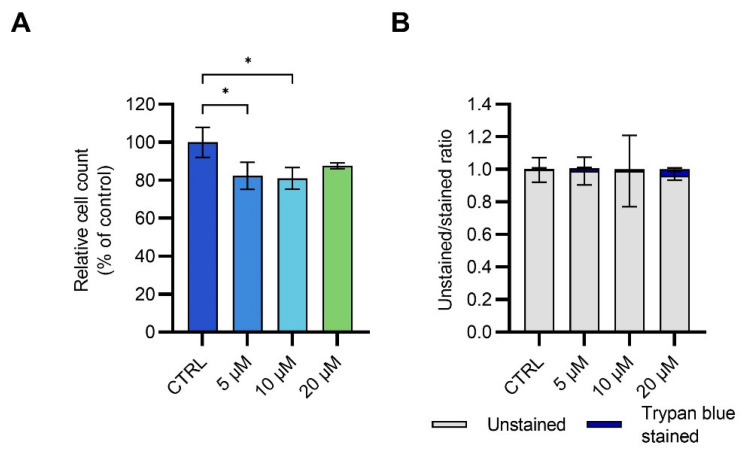
(**A**) Relative cell counts and (**B**) the ratio between live (unstained) and dead (stained) cells after 24-h treatment of HEK293T cells with 1-nitropyrene (1-nP); the plots are constructed based on cell counting. Every experiment was performed in triplicate. Data are shown as mean ± SD; statistically significant differences are marked with an asterisk: 0.01 ≤ *p* < 0.05 (*).

We also investigated cell proliferation and death upon exposure to low 1-nP concentrations (all below the determined IC_50_). Relative cell counts and the ratio between live and dead cells after the treatment with 5, 10, and 20 µM 1-nP are shown in Figure 5. Interestingly, in these experiments, almost no dead cells were observed. This implies that after the exposure to 1-nP, cell death is only provoked at high concentrations; however, DNA damage and mutations are possible even at lower concentrations [17].

## 4. Conclusions

In this work, the recently developed TD/GC-MS method for the direct analysis of atmospheric particulate PAHs and NPAHs was successfully applied to a large set of seasonal PM samples. Airborne concentrations of three NPAHs (1-nP, 9-nA, and 6-nC) attached to PM_2.5_, which is the vector for their delivery deep into the human lungs, were measured in an urban environment of Ljubljana, Slovenia and compared with PAH levels at the same destination. Roughly, NPAH concentrations were for an order of magnitude lower than the measured concentrations of their analogous PAHs in all seasons, peaking in the cold part of the year. Diagnostic ratios were further applied to apportion sources of air pollution with particulate PAHs and NPAHs, confirming incomplete combustion as the main emission source with the predomination of traffic exhausts, especially in the warmer part of the year. In the colder months, however, mixed combustion sources with non-negligible contributions of residential heating were also identified.

Cytotoxicity of four NPAHs (1-nP, 9-nA, 6-nC, and 6-nBaP) to the human kidney cell line (HEK293T) was evaluated. Among the studied pollutants, 1-nP turned out to be most potent, exhibiting moderate cytotoxic activity (IC_50_ = 28.7 µM), followed by the other three NPAHs, whose IC_50_ were above 400 µM (9-nA) and 800 µM (6-nC and 6-nBaP), demonstrating the lack of cytotoxic activity on the HEK293T cell line. The determined IC_50_ for 1-nP roughly agrees with the literature data for the HUVEC cell line, whereas no studies on human cell lines have been found for the other three analytes. It is worth noting, however, that pollutant concentrations in different tissues do not directly correlate with airborne concentrations but vary considerably depending on the type of exposure as well as the potential for accumulation. Therefore, despite its sometimes lower airborne concentrations, we evaluate 1-nP most harmful among the investigated NPAHs based on the presented cytotoxicity assessment.

Although very low airborne concentrations of NPAHs have been determined in most air-quality campaigns, their toxic potential on living organisms, especially humans, upon exposure to the polluted air is generally accepted because of the connected harmful effects on human health. Especially when bound to the smallest PM fraction, limited solubility of NPAHs in the lung fluid does not control their bioaccessibility anymore, as ultrafine particles can penetrate membranes and enter the circulatory system directly [43]. However, even when considering PM_2.5_, prolonged exposure of particulate NPAHs is expected compared with their gas-phase analogues, increasing the time for absorption and toxic effects of these poorly soluble pollutants. Therefore, besides regular air-quality monitoring and identification of relevant pollution sources, systematic toxicological assessment of NPAHs and their mixtures at different trophic levels, including cytotoxicity testing as the initial evaluation, combined with epidemiological studies on toxic effects of these pollutants is necessary in order to accurately evaluate their threat and adopt appropriate abatement strategies. Further studies are needed to observe the effect of different NPAHs on other cell types, different tissues, and whole organisms.

## Figures and Tables

**Table 1:** Seasonal concentrations (median (min–max) for every analyte) of PAHs and NPAHs in ambient PM_2.5_ from Ljubljana, Slovenia: summer 3 August–17 August 2020 and 21 June–4 July 2021; autumn 9 November–22 November 2020; winter 15 February–28 February 2021; spring 24 May–6 June 2021.

	Target Compound	Abr	Summer, ng m^−3^	Autumn, ng m^−3^	Winter, ng m^−3^	Spring, ng m^−3^
**PAH**	Acenaphthylene	Ace	n.d.	0.031 (0.004–0.109)	0.038 (0.001–0.064)	n.d.
	Fluorene	Flu	0.003 (0.001–0.011)	n.d.	n.d.	0.002 (0.001–0.010)
	Phenanthrene	Phe	0.008 (0.000–0.065)	0.107 (0.006–0.262)	0.135 (0.022–0.207)	0.016 (0.010–0.081)
	Anthracene	Ant	0.005 (0.003–0.019)	0.008 (0.002–0.033)	0.005 (0.002–0.032)	0.004 (0.002–0.009)
	Pyrene	Pyr	0.020 (0.010–0.105)	0.519 (0.091–2.788)	0.764 (0.190–1.917)	0.038 (0.006–0.137)
	Benzo(a)anthracene	BaA	0.030 (0.007–0.241)	1.119 (0.117–2.979)	0.868 (0.110–2.239)	0.057 (0.008–0.242)
	Chrysene	Cry	0.010 (0.001–0.138)	1.518 (0.192–5.711)	1.408 (0.278–2.776)	0.020 (0.001–0.116)
	Benzo(b)fluoranthene	BbF	0.044 (0.007–0.627)	1.063 (0.062–2.490)	1.476 (0.351–2.316)	0.115 (0.022–0.488)
	Benzo(k)fluoranthene	BkF	0.045 (0.002–0.691)	1.372 (0.291–2.764)	1.701 (0.201–2.361)	0.122 (0.010–0.533)
	Benzo(a)pyrene	BaP	0.026 (0.002–0.264)	1.088 (0.178–2.538)	0.987 (0.016–2.085)	0.028 (0.004–0.219)
	Indeno(1,2,3-cd)pyrene	Ind	0.035 (0.004–0.433)	0.742 (0.143–1.779)	0.646 (0.009–2.004)	0.041 (0.013–0.329)
	Dibenz(a,h)anthracene	Dba	0.016 (0.002–0.167)	0.232 (0.056–0.819)	0.264 (0.028–0.540)	0.013 (0.003–0.076)
	Benzo(g,h,i)perylene	Bgp	0.048 (0.005–0.377)	0.576 (0.134–1.597)	0.391 (0.011–1.375)	0.067 (0.014–0.344)
**NPAH**	9-nitroanthracene	9-nA	0.004 (0.002–0.007)	0.076 (0.028–0.204)	0.138 (0.029–0.334)	n.d.
	1-nitropyrene	1-nP	0.009 (0.001–0.034)	0.059 (0.025–0.184)	0.071 (0.017–0.159)	n.d.
	6-nitrochrysene	6-nC	0.007 (0.001–0.057)	0.128 (0.051–0.588)	0.096 (0.030–0.203)	0.005 (0.001–0.053)
	ΣPAH		0.208	8.807	9.234	0.516
			(0.043–3.122)	(1.516–15.285)	(1.518–15.831)	(0.050–2.584)
	ΣNPAH		0.008	0.284	0.347	0.005
			(0.001–0.063)	(0.153–0.706)	(0.079–0.545)	(0.001–0.053)
	ΣNPAH/ΣPAH		0.022	0.033	0.044	0.008
	ΣNPAH/ΣPAH_parent_		0.148	0.159	0.178	0.071
	Σ(PAH + NPAH)		0.211	9.121	9.741	0.519
			(0.049–3.139)	(1.875–15.728)	(1.597–16.300)	(0.055–2.637)

n.d.—not detected; ΣNPAH/ΣPAH_parent_ = Σ (9-nA + 1-nP + 6-nC)/Σ (Ant + Pyr + Cry).

## Data Availability

The data presented in this study are available on request from the corresponding author.

## Supplementary Materials

The following supporting information can be downloaded at: https://www.mdpi.com/article/10.3390/toxics11060518/s1, Figure S1: Particulate PAH and NPAH concentration profiles in Ljubljana, Slovenia; Figure S2: Time series of particulate PAH and NPAH concentrations for selected PAH/NPAH pairs; Figure S3: Correlations between particulate PAHs and NPAHs, and different black carbon fractions; Figure S4: IC_50_ plots for the HEK293T293 cells treatment with the selected NPAHs; Figure S5: Dead cell counts after the treatment of HEK293T293T cells with the selected NPAHs; Table S1: Concentrations of NPAHs used in the cytotoxicity testing; Table S2: Particulate PAH and NPAH concentrations in ambient air of Ljubljana, Slovenia.
