# Supplementary material for: Seasonality of Polyaromatic Hydrocarbons (PAHs) and Their Derivatives in PM2.5 from Ljubljana, Combustion Aerosol Source Apportionment, and Cytotoxicity of Selected Nitrated Polyaromatic Hydrocarbons (NPAHs)
Source: Toxics. 2023 Jun 8;11(6):518. doi: 10.3390/toxics11060518 (PMC10302792; doi:10.3390/toxics11060518)
Supplement: Supplementary file 1 [file toxics-11-00518-s001.zip › toxics-2320182-supplementary.pdf]

SUPPORTING INFORMATION FOR

Seasonality of Polyaromatic Hydrocarbons (PAHs) and Their Derivatives in $PM_{2.5}$ from Ljubljana, Combustion Aerosol Source Apportionment, and Cytotoxicity of Selected Nitrated Polyaromatic Hydrocarbons (NPAHs)

Ivana Drventić[1,2], Mateo Glumac[3], Ivana Carev[4,5,6], Ana Kroflič[7,]*

1 Department of Analytical Chemistry, National Institute of Chemistry, Hajdrihova 19, 1000 Ljubljana, Slovenia; ivana.drventic@ki.si
2 Faculty of Chemistry and Chemical Technology, University of Ljubljana, Večna pot 113, 1000 Ljubljana, Slovenia
3 Laboratory for Cancer Research, School of Medicine, University of Split, Šoltanska 2, 21000 Split, Croatia; mateo.glumac@mefst.hr
4 NAOS Institute of Life Science, 355 rue Pierre-Simon Laplace, 13290 Aix-en-Provence, France; carev.ivana@gmail.com
5 Mediterranean Institute for Life Science, Meštrovićevo šetalište 45, 21000 Split, Croatia
6 Faculty of Science, University of Split, Ruđera Boškovića 33, 21000 Split, Croatia
7 Department of Catalysis and Chemical Reaction Engineering, National Institute of Chemistry, Hajdrihova 19, 1000 Ljubljana, Slovenia
** Correspondence: ana.kroflic@ki.si; Tel.: +386-1-476-05-15*

Table S1: Tested analyte concentrations in a cell growth medium; sufficient amount of a standard in DMSO was added to the cell culture to obtain these values.

	Control	Concentrations tested (µmol l^{-1})
1-nitropyrene (1-nP)	Water with DMSO	50, 100, 200
9-nitroanthracene (9-nA)	Water with DMSO	100, 200, 400
6-nitrochrysene (6-nC)	Water with DMSO	200, 400, 800
6-nitrobenzo[a]pyrene (6-nBaP)	Water with DMSO	200, 400, 800

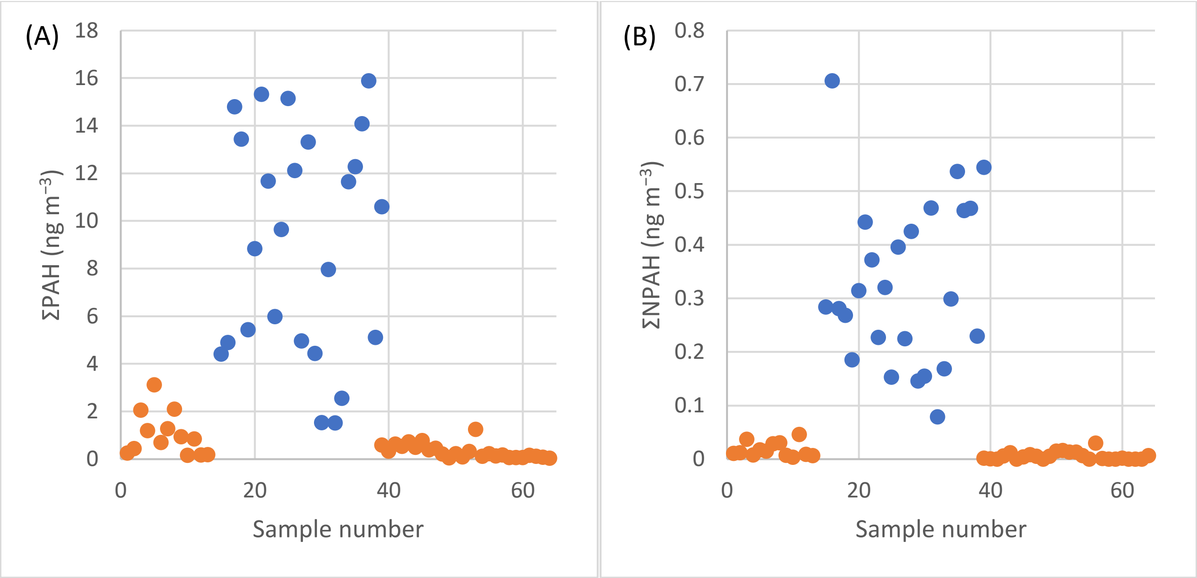

*Figure S1. (**A**) Cumulative particulate PAH and (**B**) NPAH concentration profiles (ΣPAH and ΣNPAH, respectively) in ambient air of Ljubljana, Slovenia for all five seasonal campaigns; sample numbers are adapted from Table S2. Cold-season samples (autumn, winter) are depicted with blue and warm-season samples (spring, summer) with orange symbols.*

Table S2: Particulate PAH and NPAH concentrations in ambient air of Ljubljana, Slovenia. The samples were collected in all four seasons: summer 03.08.-17.08.2020 and 21.6.-04.07.2021; autumn 09.11.-22.11.2020; winter 15.02.-28.02.2021; spring 24.05.-06.06.2021.

No.	Date	Acy [ng/m3]	Ant [ng/m3]	Phe [ng/m3]	Flu [ng/m3]	Pyr [ng/m3]	Cry [ng/m3]	BaA [ng/m3]	BbF [ng/m3]	BkF [ng/m3]	BaP [ng/m3]	DBA [ng/m3]	BgP [ng/m3]	Ind [ng/m3]	9nA [ng/m3]	1nP [ng/m3]	6nC [ng/m3]
		**Summer 2020**															
1	03/08	n.d.	0.0102	0.0108	0.00385	0.0284	0.0265	0.0766	0.0827	0.0849	0.0179	0.0231	0.0484	0.0277	0.00655	n.d.	0.0566
2	04/08	n.d.	0.0050	0.00202	0.000356	0.0156	0.0142	0.0575	0.0445	0.0515	0.00815	0.0130	0.0233	0.0155	0.00200	n.d.	0.00870
3	05/08	n.d.	0.00579	0.00544	0.000696	0.0316	0.0205	0.0493	0.0947	0.0879	0.0231	0.0164	0.0586	0.0427	0.00311	n.d.	0.00853
4	06/08	n.d.	0.0159	0.0581	0.00830	0.104	0.0768	0.150	0.249	0.646	0.143	0.0538	0.360	0.191	n.d.	n.d.	0.0372
5	07/08	n.d.	0.0102	0.0653	0.00646	0.0958	0.0348	0.106	0.194	0.200	0.0918	0.0249	0.251	0.112	0.00458	0.00307	n.d.
6	08/08	n.d.	0.0192	0.0529	0.00564	0.105	0.138	0.241	0.627	0.691	0.2644	0.167	0.377	0.433	0.00504	0.0122	n.d.
7	09/08	n.d.	0.0117	0.0418	0.0106	0.0665	0.0250	0.0740	0.134	0.126	0.0290	0.0184	0.0866	0.0690	0.00390	0.0112	n.d.
8	11/08	n.d.	0.00900	0.0148	0.00190	0.0405	0.0646	0.106	0.247	0.337	0.116	0.0629	0.128	0.152	0.00507	0.00963	0.0137
9	12/08	n.d.	0.0120	0.0308	0.00270	0.0725	0.121	0.212	0.454	0.413	0.183	0.117	0.240	0.236	0.00332	0.0272	n.d.
10	13/08	n.d.	0.00821	0.0232	0.00290	0.0521	0.0556	0.104	0.186	0.199	0.0634	0.0411	0.115	0.0839	0.00496	0.00222	n.d.
11	14/08	n.d.	0.00526	0.00634	0.00169	0.0204	0.00889	0.0345	0.0325	0.0279	n.d.	0.00579	0.0161	0.00436	0.00324	n.d.	0.000519
12	15/08	n.d.	0.00865	0.0229	0.00295	0.0342	0.0413	0.0771	0.185	0.159	0.0620	0.0453	0.0999	0.106	0.00273	0.0342	0.00879
13	16/08	n.d.	0.00575	0.00273	n.d.	0.0150	0.00816	0.0231	0.0372	0.0343	0.00187	0.00859	0.0211	0.0159	0.00356	n.d.	0.00507
14	17/08	n.d.	0.00393	0.00468	0.000222	0.0239	0.0114	0.0329	0.0427	0.0369	0.003017	0.00412	0.0161	0.00655	0.00172	n.d.	0.00487
		**Autumn 2020**															
15	12/11	n.d.	0.00347	n.d.	n.d.	0.238	0.500	0.316	0.916	0.824	0.532	0.169	0.396	0.520	0.0311	0.0367	0.216
16	13/11	0.0598	0.00523	0.193	0.00172	1.08	2.03	n.d.	0.0617	n.d.	n.d.	0.234	0.535	0.6911	0.0839	0.0334	0.588
17	14/11	0.0660	0.00859	0.112	n.d.	0.860	2.19	1.54	1.288	2.76	2.54	0.800	1.13	1.49	0.204	0.0252	0.0513
18	15/11	0.0421	0.00412	0.0833	n.d.	0.775	2.27	1.568	1.81	2.68	n.d.	0.819	1.60	1.78	0.132	0.0246	0.112
19	16/11	0.00440	0.00388	n.d.	n.d.	0.245	0.497	0.334	1.06	0.992	n.d.	0.396	0.851	1.04	0.0478	0.0742	0.0632
20	17/11	0.0250	0.0122	0.0362	n.d.	0.519	1.34	1.06	1.74	1.56	1.34	0.219	0.489	0.508	0.0877	0.0763	0.150
21	18/11	0.0305	0.0100	0.101	n.d.	1.36	2.67	2.28	2.49	2.40	2.04	0.299	0.698	0.950	0.0809	0.184	0.178
22	19/11	0.0322	0.0307	0.165	2.96E-05	1.05	1.90	1.41	1.80	2.19	1.60	0.229	0.466	0.792	0.0593	0.0980	0.214
23	20/11	0.0138	0.00785	n.d.	n.d.	0.408	0.667	0.456	1.06	1.08	0.822	0.228	0.576	0.662	0.0399	0.0589	0.128
24	21/11	0.0188	0.00407	n.d.	n.d.	0.495	1.52	1.18	1.61	1.52	1.36	0.373	0.711	0.847	0.0837	0.169	0.0670
25	22/11	0.1089	0.0171	0.262	n.d.	2.79	5.71	2.98	0.524	1.22	0.840	n.d.	0.687	n.d.	n.d.	0.0315	0.121

		**Winter 2021**															
26	15/02	0.0292	0.0325	0.168	n.d.	0.895	1.02	0.660	1.76	1.77	1.86	0.540	1.37	2.00	0.0809	0.159	0.156
27	16/02	0.0111	0.0162	0.0524	n.d.	0.560	0.514	0.266	0.696	0.591	0.416	0.238	0.733	0.867	0.0903	0.0658	0.0688
28	17/02	0.0432	0.0116	0.0944	n.d.	1.31	2.77	2.24	2.02	1.94	1.23	0.298	0.602	0.765	0.238	0.0880	0.0994
29	18/02	0.0161	0.00819	0.0423	n.d.	0.496	0.572	0.324	0.934	0.723	0.295	0.157	0.383	0.486	0.0556	0.0604	0.0297
30	19/02	0.000756	0.00462	n.d.	n.d.	0.204	0.385	0.147	0.436	0.201	0.0212	0.0306	0.0375	0.0642	0.0806	0.0744	n.d.
31	20/02	0.0333	0.00153	0.0215	n.d.	0.633	1.83	1.08	1.82	2.05	0.461	0.0281	0.0149	n.d.	0.334	0.0432	0.0909
32	21/02	n.d.	0.00203	n.d.	n.d.	0.190	0.278	0.110	0.351	0.534	0.0158	n.d.	n.d.	0.0369	0.0290	0.0165	0.0331
33	22/02	n.d.	0.00434	n.d.	0.00181	0.247	0.599	0.263	0.510	0.645	0.258	n.d.	0.0106	0.00927	0.0644	0.104	n.d.
34	23/02	0.0636	0.00240	0.158	0.00133	1.27	2.52	1.81	1.49	1.93	1.70	0.0811	0.217	0.386	0.185	0.0308	0.0831
35	24/02	0.0618	0.00458	0.207	n.d.	1.45	2.24	1.63	1.73	1.65	1.96	0.314	0.391	0.646	0.262	0.0716	0.203
36	25/02	0.0542	0.00381	0.170	n.d.	1.92	2.58	1.97	1.96	1.99	2.09	0.288	0.373	0.687	0.288	0.0710	0.105
37	26/02	0.0534	0.00410	0.150	n.d.	1.48	2.36	1.94	2.32	2.36	2.02	0.508	1.27	1.42	0.238	0.138	0.0917
38	27/02	0.0127	0.00730	n.d.	n.d.	0.360	0.562	0.337	0.728	0.898	0.747	0.240	0.615	0.605	0.0344	0.0370	0.158
39	28/02	0.0618	0.0265	0.121	0.00329	1.05	1.80	1.23	1.46	1.75	1.25	0.3358	0.742	0.770	0.286	0.151	0.108
		**Spring 2021**															
40	26/05	n.d.	0.00348	0.0143	0.00142	0.0376	0.0235	0.0634	0.124	0.142	0.0310	0.0168	0.0768	0.0572	n.d.	n.d.	0.00190
41	27/05	n.d.	0.00333	0.0157	0.00144	0.0314	0.0121	0.0385	0.0670	0.0633	0.0123	0.00867	0.0447	0.0304	n.d.	n.d.	0.000578
42	28/05	n.d.	0.00372	0.0120	0.000578	0.0511	0.0282	0.0573	0.127	0.155	0.0370	0.0154	0.0731	0.0681	n.d.	n.d.	n.d.
43	29/05	n.d.	0.00393	0.0161	0.00156	0.0411	0.0192	0.0537	0.120	0.132	0.0284	0.0134	0.0604	0.0412	n.d.	n.d.	0.00606
44	30/05	n.d.	0.00510	0.0249	0.00287	0.0552	0.0308	0.0766	0.150	0.172	0.0490	0.0192	0.0723	0.0642	n.d.	n.d.	0.0121
45	31/05	n.d.	0.00239	0.0203	0.000430	0.0467	0.0231	0.0656	0.109	0.113	0.0267	0.0112	0.0447	0.0390	n.d.	n.d.	n.d.
46	01/06	n.d.	0.00237	0.0312	0.00390	0.0498	0.0312	0.0868	0.159	0.175	0.0430	0.0241	0.095	0.0739	n.d.	n.d.	0.00391
47	02/06	n.d.	0.00317	0.0217	0.00290	0.0313	0.0173	0.0567	0.0901	0.0789	0.0169	0.00862	0.0365	0.0278	n.d.	n.d.	0.00836
48	03/06	n.d.	0.00364	0.0105	0.00185	0.0294	0.0166	0.0486	0.111	0.0989	0.0196	0.0109	0.0667	0.0365	0.00483	n.d.	0.000237
49	04/06	n.d.	0.00270	n.d.	n.d.	0.0128	0.0121	0.0368	0.0600	0.0591	0.00390	0.00514	0.0143	0.0134	n.d.	n.d.	n.d.
50	05/06	n.d.	0.00295	n.d.	n.d.	0.00573	0.00136	0.00837	0.0219	0.00981	n.d.	n.d.	n.d.	n.d.	0.00396	n.d.	0.00114
51	06/06	n.d.	0.00647	0.0145	0.000933	0.0205	0.00782	0.0237	0.0423	0.0514	0.00653	0.00333	0.030	0.0198	n.d.	n.d.	0.0150
		**Summer 2021**															
52	21/06	n.d.	0.00304	0.00871	0.00166	0.0137	0.00239	0.0148	0.0205	0.0106	n.d.	0.00409	0.0140	0.000163	n.d.	0.0149	0.00145
53	22/06	n.d.	0.00489	0.0141	0.00364	0.0229	0.00778	0.0273	0.0501	0.0439	0.0104	0.00530	0.104	0.0215	n.d.	0.00449	0.00858

54	23/06	n.d.	0.00756	0.0251	0.00259	0.0484	0.0552	0.108	0.285	0.276	0.0859	0.0564	0.157	0.136	n.d.	0.00862	0.00427
55	24/06	n.d.	0.00280	0.00959	0.000311	0.0163	0.00425	0.0215	0.0288	0.0192	n.d.	0.00182	0.0121	0.00418	n.d.	0.00493	0.00139
56	25/06	n.d.	0.00375	0.01159	0.000622	0.0178	0.00720	0.0243	0.0496	0.0471	0.00704	0.00625	0.0317	0.0226	n.d.	n.d.	n.d.
57	26/06	n.d.	0.00373	0.00560	0.00236	0.00956	0.00344	0.0116	0.0341	0.0274	n.d.	0.00504	0.0201	0.0152	n.d.	n.d.	0.0298
58	27/06	n.d.	0.00402	0.00270	0.00206	0.0118	0.00356	0.0120	0.0400	0.0357	0.000385	0.00956	0.0304	0.0263	n.d.	0.00101	n.d.
59	28/06	n.d.	0.00262	4.44E-05	n.d.	0.0110	0.00150	0.00994	0.0142	0.00980	n.d.	0.000178	0.0204	n.d.	n.d.	n.d.	n.d.
60	29/06	n.d.	0.00324	0.00258	n.d.	0.0127	0.00379	0.0132	0.0180	0.0132	n.d.	n.d.	n.d.	n.d.	n.d.	n.d.	n.d.
61	30/06	n.d.	0.00350	0.00578	0.000859	0.0112	0.00194	0.0122	0.0176	0.0119	n.d.	n.d.	0.00495	n.d.	n.d.	n.d.	0.00154
62	01/07	n.d.	0.00311	0.006963	0.000370	0.0198	0.00813	0.0188	0.0428	0.0461	0.00692	n.d.	n.d.	4.44E-05	n.d.	n.d.	n.d.
63	02/07	n.d.	0.00324	0.00421	n.d.	0.0150	0.0109	0.0211	0.0310	0.0288	0.00207	n.d.	n.d.	n.d.	n.d.	n.d.	n.d.
64	03/07	n.d.	0.003350	0.00489	0.000104	0.0113	0.00268	0.0117	0.0239	0.0147	n.d.	n.d.	n.d.	n.d.	n.d.	n.d.	n.d.
65	04/07	n.d.	0.00563	0.00655	0.000578	0.0116	0.00212	0.00701	0.00717	0.00239	n.d.	n.d.	n.d.	n.d.	n.d.	n.d.	0.00630

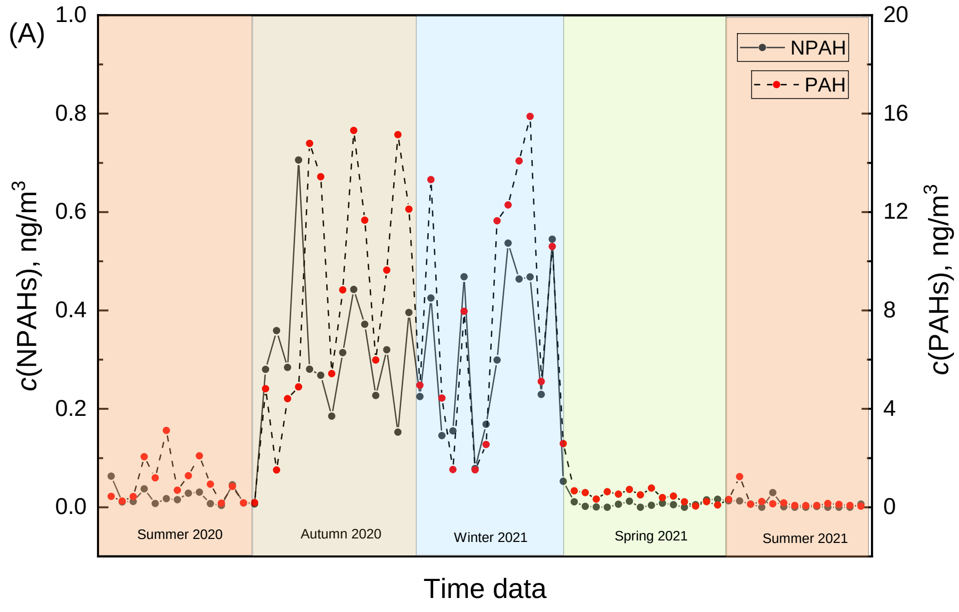
(A)
NPAH
PAH
c(NPAHs), ng/m3
c(PAHs), ng/m3
Summer 2020
Autumn 2020
Winter 2021
Spring 2021
Summer 2021
Time data

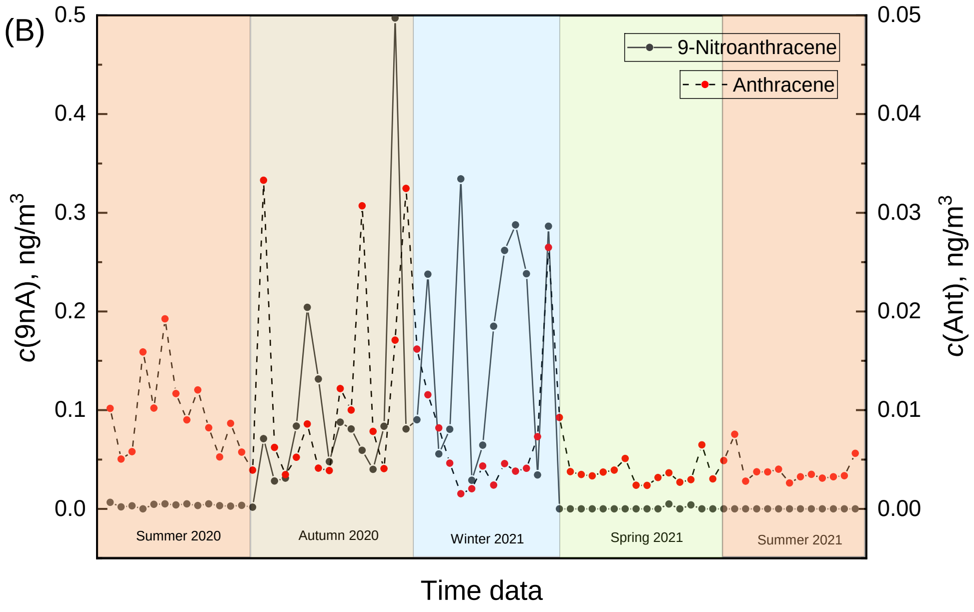
(B)
9-Nitroanthracene
Anthracene
c(9nA), ng/m3
c(Ant), ng/m3
Summer 2020
Autumn 2020
Winter 2021
Spring 2021
Summer 2021
Time data

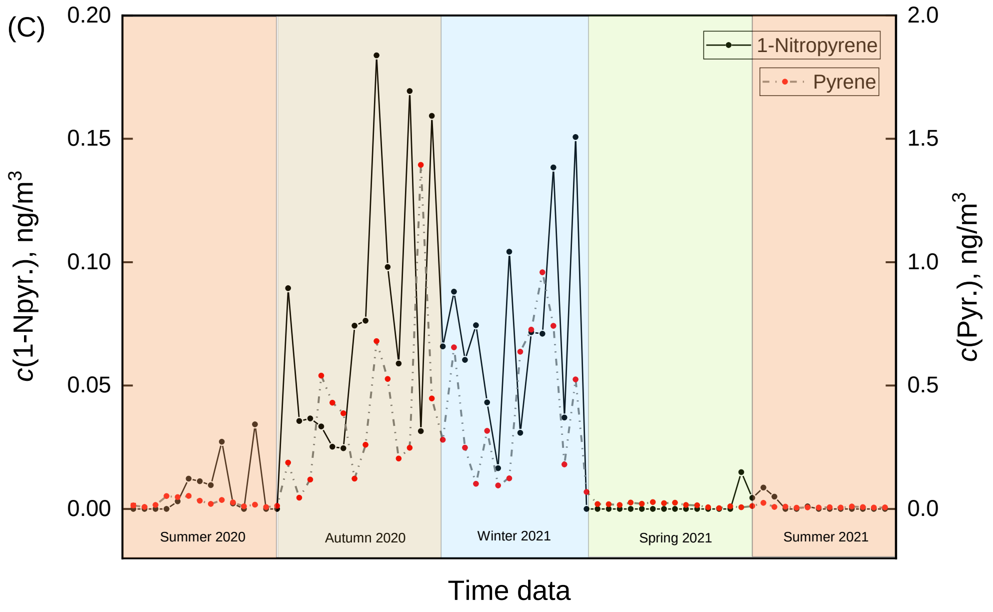

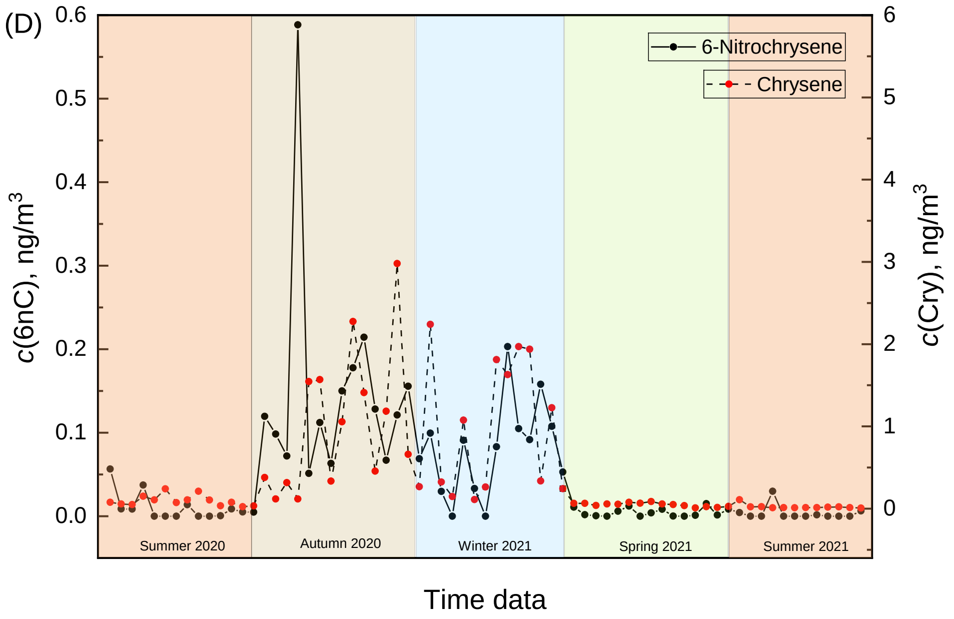

*Figure S2. Time series of particulate PAH and NPAH concentrations for selected PAH/NPAH pairs: (**A**) ΣPAH and ΣNPAH, (**B**) anthracene (Ant) and 9-nitroanthracene (9nA), (**C**) pyrene (Pyr) and 1-nitropyrene (1nP), and (**D**) chrysene (Cry) and 6-nitrochrysene (6nC).*

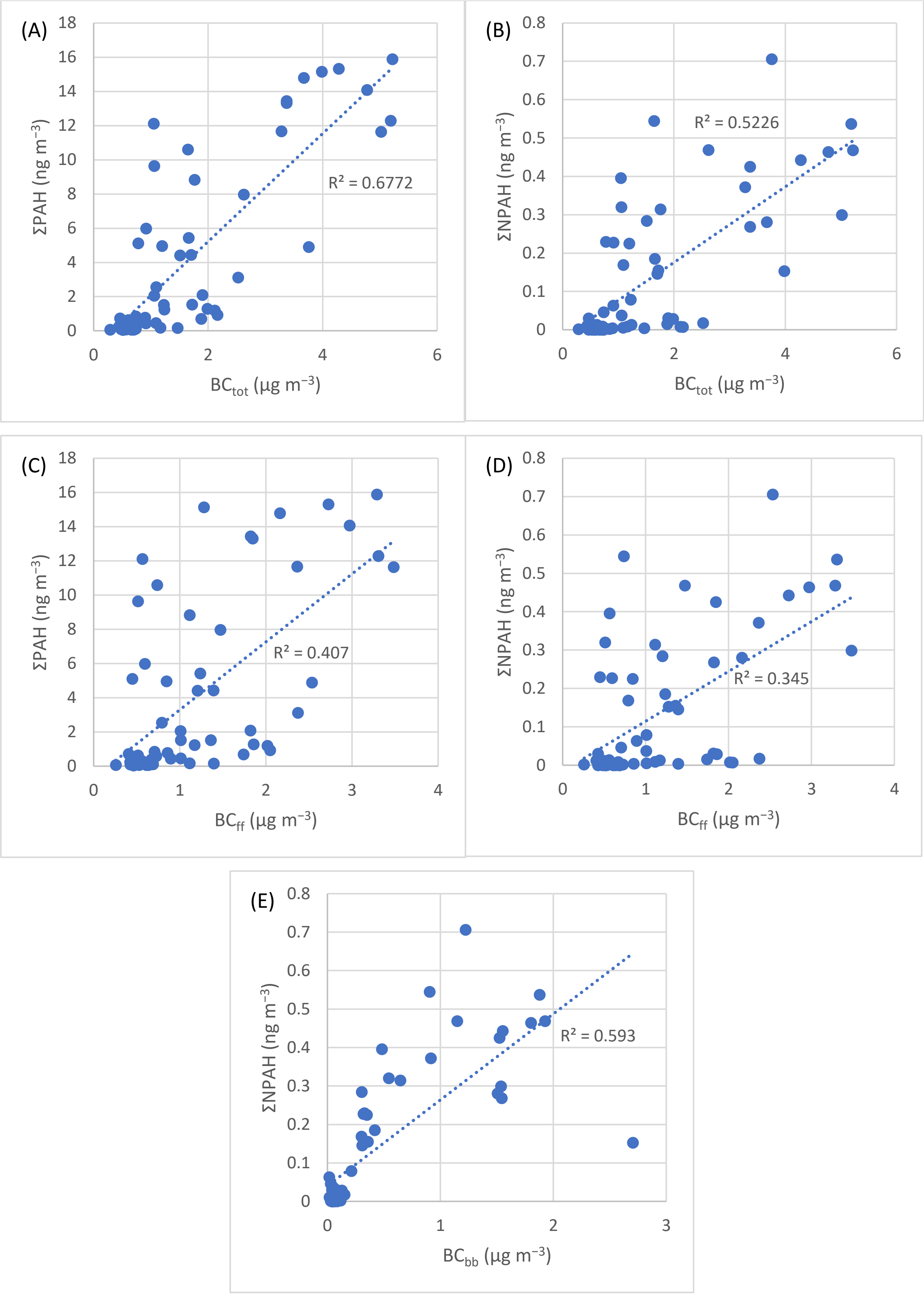

*Figure S3. Correlations between cumulative particulate PAHs and NPAHs (ΣPAH and ΣNPAH), and (**A,B**) total black carbon (BC_{tot}), (**C,D**) black carbon from traffic (BC_{ff}), and (**E**) black carbon from biomass burning (BC_{bb}).*

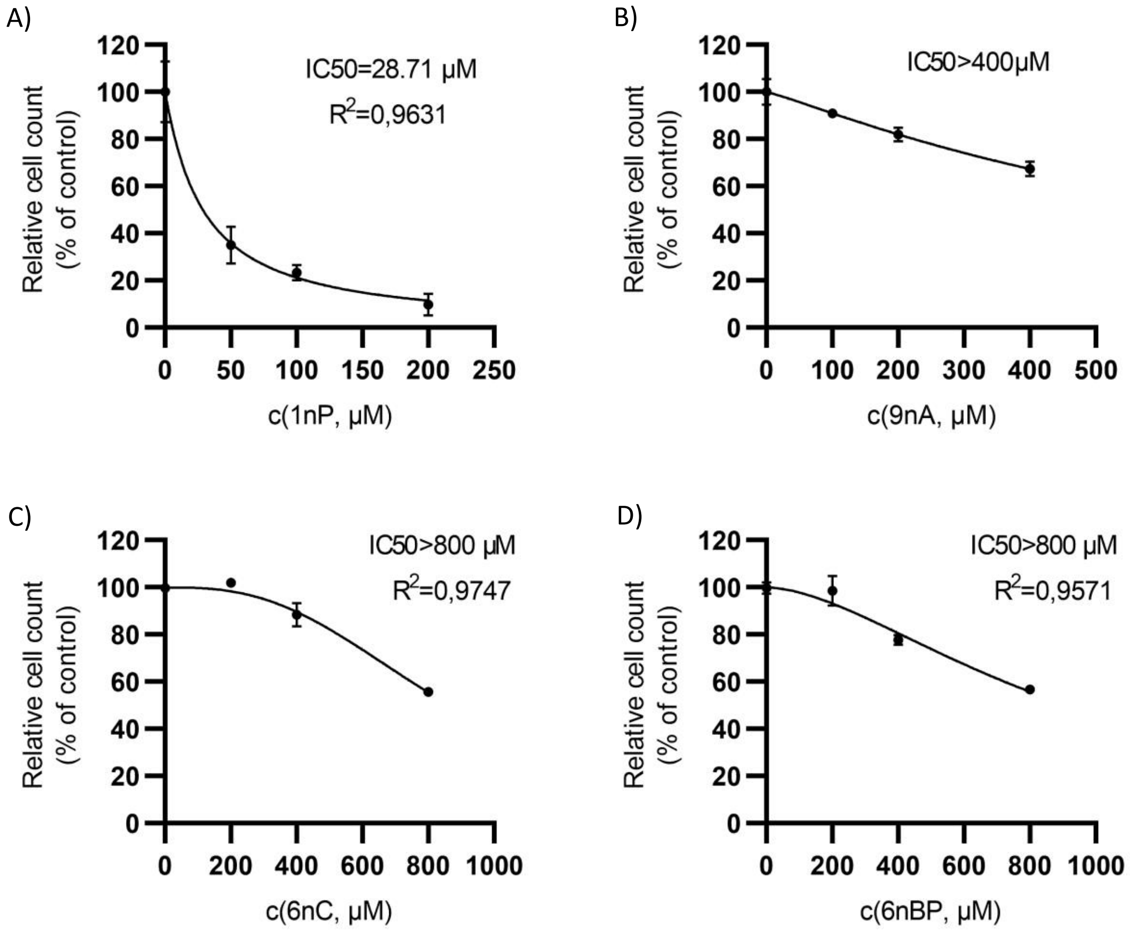

*Figure S4. IC_{50} plots for 24-hours treatment of HEK293T cells with (**A**) 1-nitropyrene (1-nP), (**B**) 9-nitroanthracene (9-nA), (**C**) 6-nitrochrysene (6-nC), and (**D**) 6-nitrobenzo[a]pyrene (6-nBaP); the plots are constructed based on cell counting. Every experiment was performed in triplicate.*

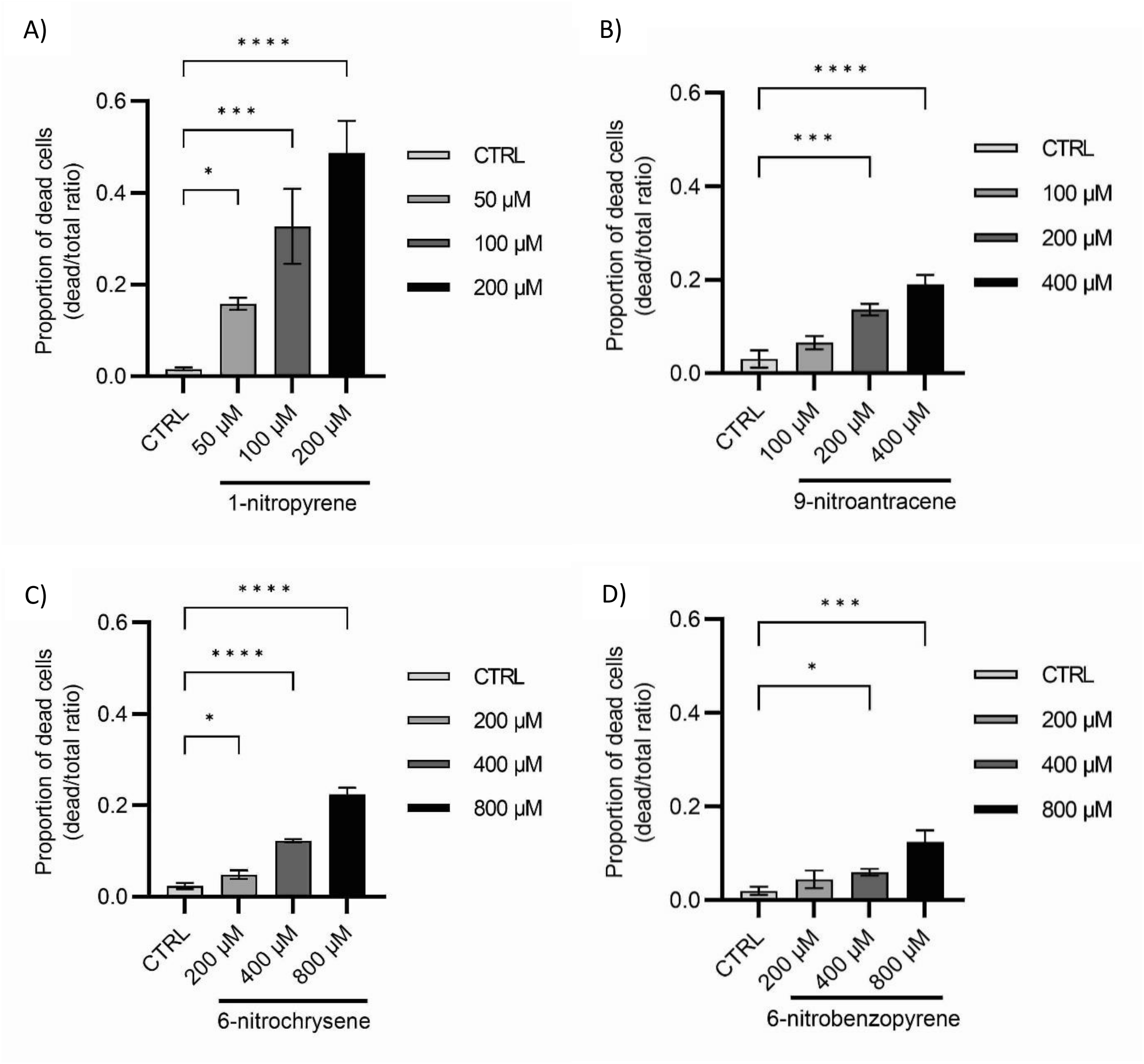

*Figure S5. Dead cell counts after 24-hours treatment of HEK293T cells with (**A**) 1-nitropyrene (1-nP), (**B**) 9-nitroanthracene (9-nA), (**C**) 6-nitrochrysene (6-nC), and (**D**) 6-nitrobenzo[a]pyrene (6-nBaP); for every treatment, control experiment (CTRL) is also given for comparison. Every experiment was performed in triplicate and statistically evaluated by Dunnett's multiple comparison test. Data are shown as mean ± SD; different significance levels are assigned with asterisks: 0.01≤p<0.05 (*), 0.001≤p<0.01 (**), 0.0001≤p<0.001 (***), p<0.0001 (****).*
